# How should implementation of the human right to health be assessed? A scoping review of the public health literature from 2000 to 2021

**DOI:** 10.1186/s12939-022-01742-0

**Published:** 2022-09-22

**Authors:** Lisa Montel, Naomi Ssenyonga, Michel P. Coleman, Claudia Allemani

**Affiliations:** 1 Cancer Survival Group, London School of Hygiene and Tropical Medicine, Keppel Street, London, WC1E 7HT UK; 2 Centre for Health, Law and Society, Faculty of Social Sciences and Law, University of Bristol, Bristol, UK; 3 Cancer Division, University College London Hospitals NHS Foundation Trust, London, UK

**Keywords:** Right to health, Human rights-based approach to health, Rights-based approach to health, Indicators, Scoping review

## Abstract

The human right to health is a critical legal tool to achieve health justice, and universal health coverage is included among the Sustainable Development Goals. However, the content and meaning of the right to health may not be used adequately in public health research. We conducted a scoping review of the literature to discover the extent to which the legal principles underlying the right to health are used in public health. We mapped the various attempts to assess implementation of this right since its legal content was clarified in 2000.

The first studies emerged in 2006, with an increase and a wider variety of investigations since 2015. We observe that some key principles do form the basis of right-to-health assessments, but some concepts remain unfamiliar. Critically, public health academics may have limited access to human rights research on health, which creates a gap in knowledge between the two disciplines.

**Supplementary Information:**

The online version contains supplementary material available at 10.1186/s12939-022-01742-0.

## Introduction

Health is a human right. Everyone, regardless of sex, religion, age, ethnicity or nationality, is entitled to health services, medicines and equipment that are available, accessible, acceptable and of good quality [1–3].

After World War II, health – along with other necessities such as education and work – came to be considered as universal rightful entitlements. In 1946, the Constitution of the World Health Organisation (WHO) affirmed that “the enjoyment of the highest attainable standard of health is one of the fundamental rights of every human being” [4]. Two years later, the right to health was enacted in the Universal Declaration of Human Rights [3]. Although these political instruments marked a clear willingness to define health as a fundamental right, it was only in 1966, with Article 12 of the International Covenant on Economic, Social and Cultural Rights (the Covenant), that implementing the right to health for all citizens became a legally binding commitment for signatory states [1]. In 1978, health was recognised as critical in maintaining peace between countries. The Alma-Ata Declaration framed health as a shared goal, with primary health care anchored in human rights principles [5].

The right to health, like other economic and social rights, is subject to the principle of “progressive realisation” [6]. According to this principle, states are required to take some immediate steps to fulfil the right to health, and must not take any retrogressive measure [2, 7]. Progressive realisation also means that each state must monitor achievement of the right to health on a regular basis, in order to assess whether it is making adequate progress, for any given level of resources.

Monitoring economic and social rights therefore requires adequate indicators that are grounded in human rights principles. The search for indicators began with the appointment of the UN Special Rapporteur on the realisation of Economic, Social and Cultural Rights, and a UN seminar in 1993 on “appropriate indicators to measure achievements in the progressive realisation of economic, social and cultural rights” [8]. However, it was agreed that the content of each right and the corresponding obligations upon member states should first be clearly defined.

The Committee on Economic, Social and Cultural Rights, the body responsible for monitoring the implementation of the Covenant of 1966, issued a formal interpretation of the right to health (General Comment 14) in 2000 [2]. General Comment 14 sets out the “normative” content of the right to health. It lists the obligations for states and the potential remedies for citizens if their rights are not respected. It incorporates many human rights principles, such as non-discrimination, participation and transparency, as well as an overarching framework on “Availability, Accessibility, Acceptability and Quality” of health services and medicines (the AAAQ framework). In 2009, WHO issued guidance on a human rights-based approach to health using General Comment 14 as a basis [9]. General Comment 14 is neither perfect nor legally binding, but it provides a shared understanding of what the right to health entails in practice [10].

With a clearer definition of the right to health, research on indicators rapidly became a new field of legal scholarship from 2000. This type of research was also developed by the first UN Special Rapporteur on the right to health, Paul Hunt, and culminated in 2012 with the adoption of a framework for the development of human rights indicators by the Office of the High Commissioner for Human Rights (OHCHR) [11].

During most of the period 1946 to 2012 covered by these developments, the public health community has carried out research on health inequities and the social determinants of health. This body of research provides empirical evidence of the interconnectedness of human rights, and of health as a social phenomenon, rather than simply the outcome of the provision of health care [12, 13]. Work on the social determinants of health complements the work of the OHCHR on the measurement of human rights at structural, process and outcome levels. Both types of research reveal the challenges that arise in monitoring realisation of the right to health: such an exercise must go beyond assessment of health inequalities to include the broader structure of power relationships, patterns of discrimination and social arrangements that can impact health.

Despite these breakthroughs in understanding of the right to health and the duties that are incumbent on states to deliver it, research in human rights and in public health has mostly been conducted in separate silos. Concepts such as non-discrimination, accountability, participation, core obligations and the progressive realisation of these human rights seem to bear a legal meaning that may be unfamiliar to epidemiologists, health systems researchers or health policymakers. We refer to these groups collectively as the “public health community” as opposed to the “human rights community”, which we use for scholars having a legal background. The vocabulary differs widely between these two communities, to the point where the same words may have different meanings [14]. Notably, the term “right to health care” is often used in the public health community to indicate the human right to heath, whereas health care is only one of several components of the right to health.

Today, the right to health is widely understood by the human rights community, and it is relied upon by civil society. To our knowledge, however, the extent to which the right to health is understood and assessed in public health has not been explored.

We conducted a scoping review to understand the extent to which the principles underlying the right to health are perceived and used in the public health community. We mapped the various attempts to assess implementation of the right to health since the normative principles were enacted in 2000 [2].

The questions that guided our review were:

1. What types of public health research on the right to health are available?
2. To which areas of public health has this research been applied?
3. What principles define the right to health?
4. Which are the most common indicators in the public health and legal fields and to what extent are they different? How well do they capture the meaning of the principles?
5. Which legal sources are relied upon to define the right to health?

## Search strategy and selection criteria

We followed the PRISMA guidelines for systematic reviews, although our work is better qualified as a “scoping review” under Grant and Booth’s typology of reviews [15, 16].

We searched five databases commonly used in public health literature: Embase, Global Health, Medline, Open Grey, and Dissertations and Theses Global. We used the keywords “right to health”, “human rights-based approach to health” and “indicators” or “framework”. The Boolean query formulations were checked and approved by a librarian at the London School of Hygiene and Tropical Medicine.

We included systematic reviews, texts and opinions, including editorials for journal issues on the right to health or human rights-based approaches to health. We excluded material that was not published in peer-reviewed journals, such as reports or guidelines from non-governmental organisations, international organisations and universities. Our review focused specifically on the right to health, as opposed to human rights more generally, because we were interested in what components of this right are assessed in public health and how. Therefore, we excluded studies that framed a health issue in human rights terms (such as the dignity of patients with HIV), as well as studies on the integration of human rights principles into health policies, programmes and guidelines, and on human rights indicators. The categories of studies included and excluded are shown in box 1 and box 2, respectively. We searched for studies published since 2000, the year when General Comment 14 was adopted. The reference lists of included studies were screened for additional relevant articles.

**Table:** 

We included studies published in English, French, Spanish or Italian between 2000 and July 2021 that:	
• Discuss the definition of the right to health and its principles;	
• Discuss one or more specific elements of the right to health (e.g., participation) or human rights-based approaches to health, while using narratives of the right to health;	
• Construct methodologies for the evaluation of the right to health;	
• Construct new indicators for the evaluation of the right to health;	
• Use public health indicators for the evaluation of the right to health;	
• Discuss public health indicators in the light of human rights principles;	
• Evaluate a health policy, programme, health outcomes or a health system, while using narratives of the right to health.	

## Data extraction

**Table:** 

We excluded studies:	
• on the rights of persons with disabilities;	
• on informed consent;	
• on the national legal system and health outcomes;	
• on the impact of human rights on health;	
• framing a health issue in human rights terms;	
• on reproductive rights;	
• on “client-centred” or “people-centred” approaches;	
• on the integration of human rights principles into health policies, programmes and guidelines;	
• on human rights indicators;	
• on economic and social rights;	
• on the links between human rights and health;	
• on rights-based approaches to development;	
• on gender equality;	
• on the social determinants of health;	
• on the Sustainable Development Goals when the focus was not the right to health;	
• on the judicialisation of the right to health.

**Table:** 

Extraction fields:	
• Year published;	
• Discipline: human rights, public health, or both;	
• Area of public health;	
• Type of research;	
• International instruments referred to;	
• Principles of the right to health.	

We referred to the authors’ background and affiliation and to the journal to determine the discipline of each study. We labelled the subject area as “both” when the authors were experts in human rights and public health.

The area of public health could be disease-oriented, such as HIV, TB and malaria; group-oriented, such as maternal and child health; or theme-oriented, such as universal health coverage or access to medicines. The “general” category included articles that did not focus on a specific disease, group or theme, but examined the right to health applied to health systems or health policy.

## Role of the funding source

None.

## Results

**Fig. 1:**
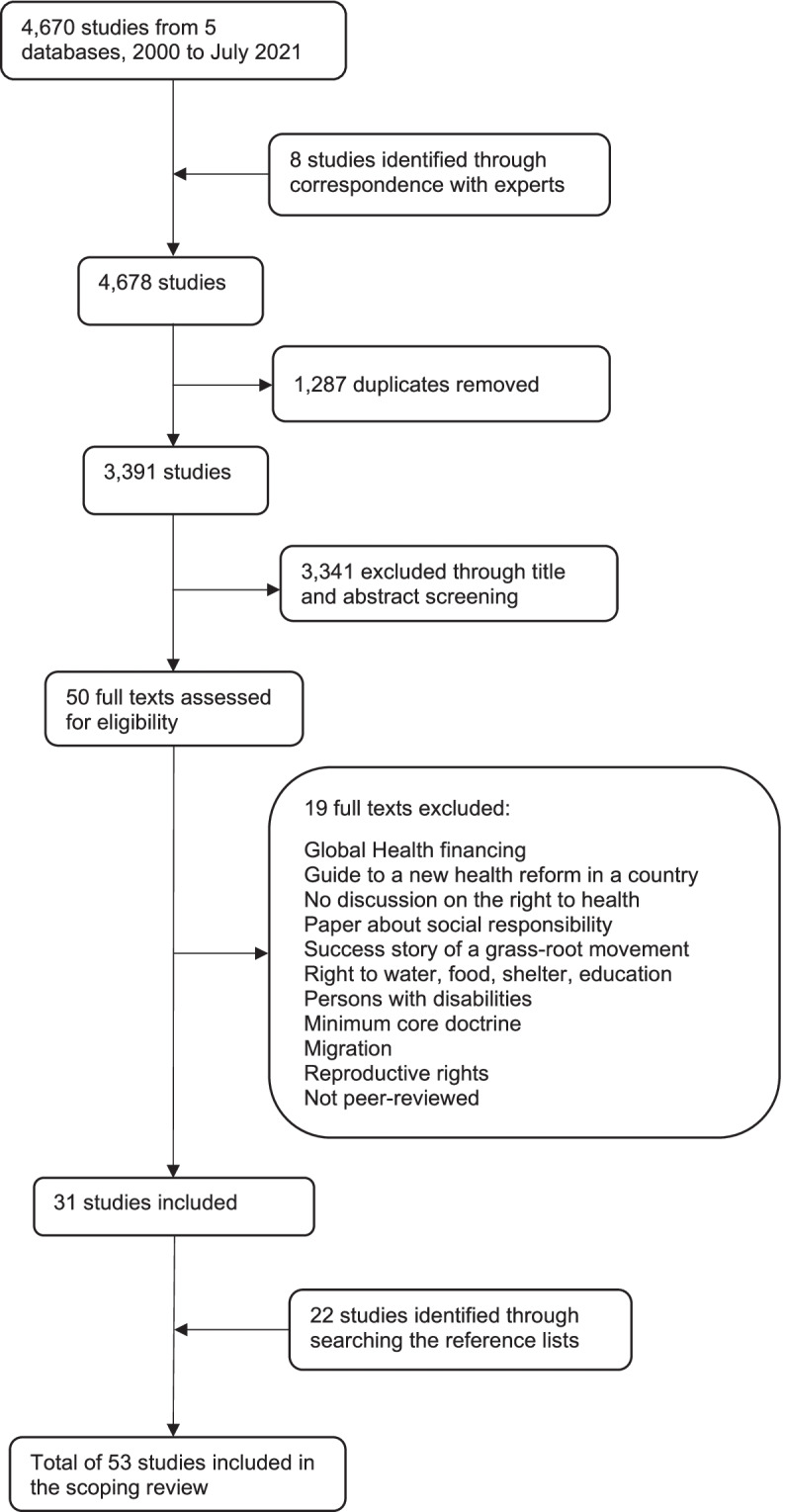
Literature search

We retrieved 4670 studies through database search and added 8 studies from correspondence with experts. After removal of duplicates and screening of titles and abstracts, we assessed the full text of 50 studies, of which 31 were included in our review. A further 22 articles were included from reference searches, 53 in total (Fig. 1). All full-text papers were published in English.

**Fig. 2:**
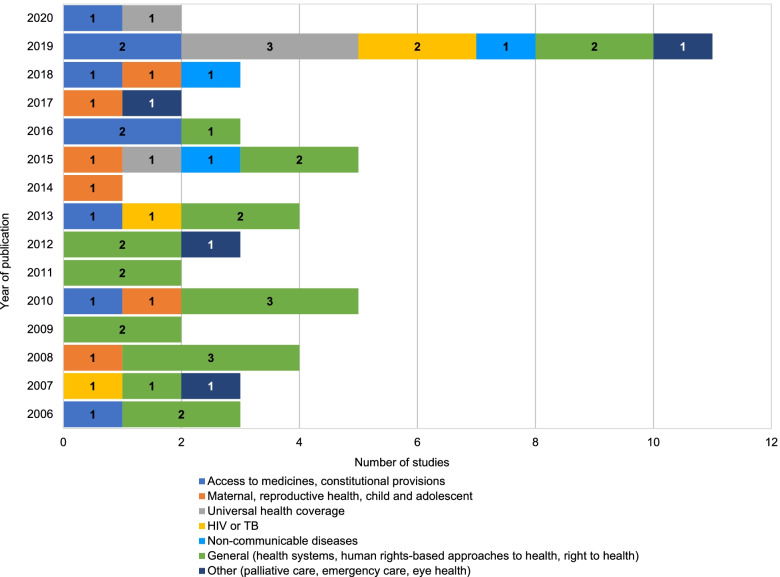
Studies by year and area of public health

There was no study on the assessment of the right to health published between 2000 and 2005 (Fig. 2). From 2006 to 2014, 27 studies were published. Most of these studies were general (17), i.e., discussions of the right to health and its principles; discussions on or proposals of new scientific methods or indicators to assess the right to health; discussions on human rights based-approaches to health; integration of right-to-health norms and values to health systems; and innovative methods to assess the right to health. Studies were mainly applied to the field of maternal, child and reproductive health (3), followed by HIV or TB (2).

We observed a slight increase from 2015, with 26 studies published in 6 years, versus 27 in the previous 9 years. Two peaks appear in the volume of studies in 2015 and 2019. Attempts to assess the right to health were applied to new fields, notably non-communicable diseases (3) and universal health coverage (5). In the meantime, the underlying trend has continued since 2015, with more general studies (5) and studies of maternal, child and reproductive health (3) and HIV or TB (2).

Reviews on access to medicines or reviews of constitutional provisions were consistently published since 2006. One landmark study in 2013 examined the status of health provisions in 191 constitutions.

Two studies focussed on palliative care as a human right (2007 and 2017) [17, 18]. Other areas of focus include eye health (2012) and emergency care (2019).

**Fig. 3:**
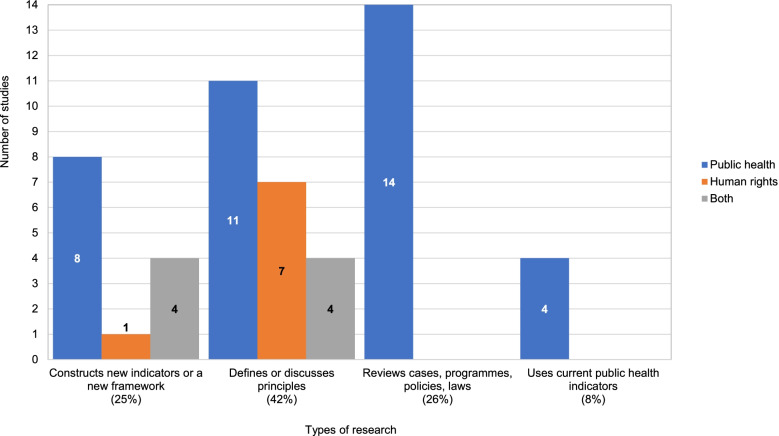
Studies by category of research and by discipline

Forty-two percent of studies define or discuss principles of the right to health with a view to clarifying what a human rights-based approach to health means in practice, or how to assess progress towards implementation of the right to health (Fig. 3). Twenty-five percent of the studies go further, constructing new frameworks to assess the implementation of the right to health, or human rights-based approaches to health. Two of these studies constructed new indicators to assess implementation of the right to health, while 11 constructed new frameworks to assess the extent to which health policies or programmes comply with human rights principles. Similarly, 26% of studies review laws, policies, programmes or cases in light of principles that underpin the right to health. The remaining studies (8%) use existing public health indicators to assess progress towards the implementation of the right to health. Details are provided in Additional file 1.

Public health scholars have published studies in all four categories, but in the articles retrieved, human rights experts have mainly focused on defining or discussing principles of the right to health.

The most comprehensive attempt to construct indicators of the implementation of the right to health, in terms of anchoring of the method in human rights principles, the number of indicators, and the diversity of areas concerned, was published in 2008 under the authorship of the first UN Special Rapporteur on the right to health [10]. Some of these indicators were used in two later studies [19, 20].

**Fig. 4:**
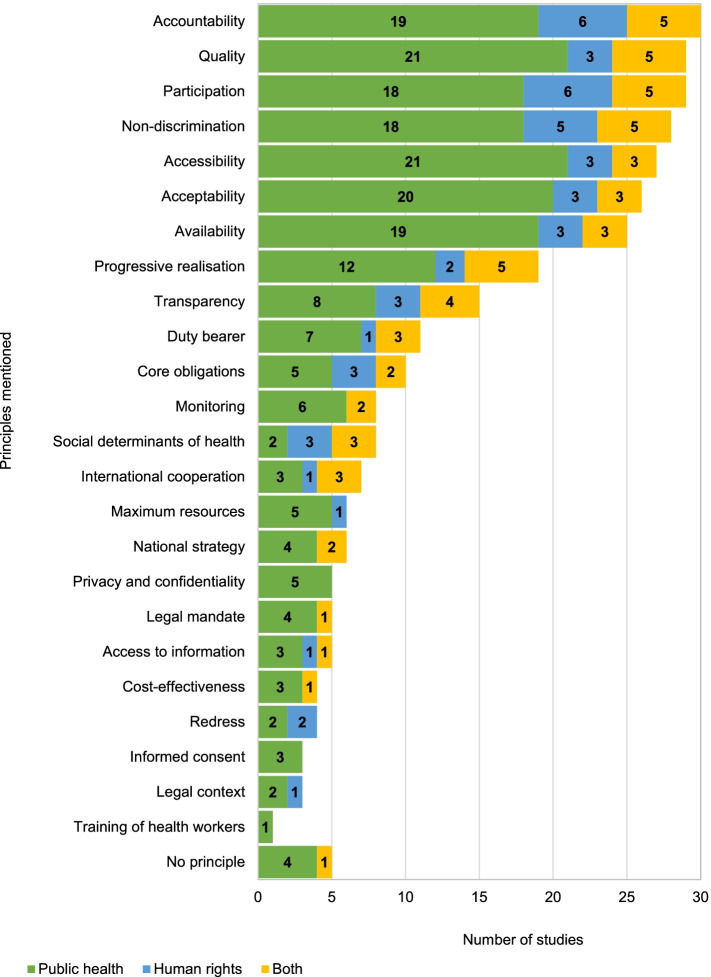
Principles of the right to health by discipline

**Fig. 5:**
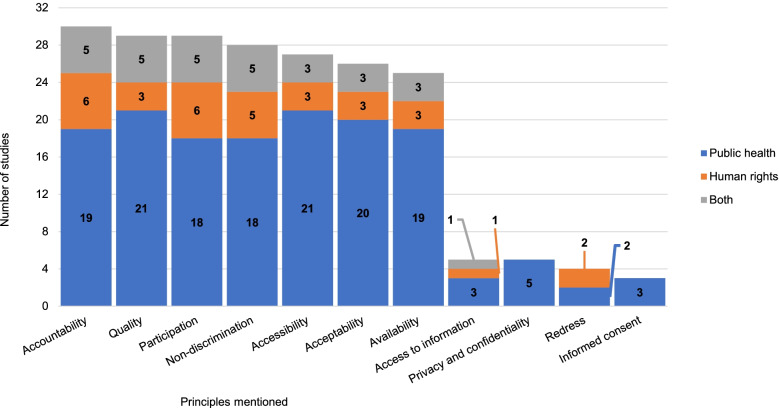
Principles commonly assessed under the right to health

We identified 24 principles of the right to health mentioned in one or more of these studies (Fig. 4). The seven principles mentioned in most studies, whether in human rights or public health, are non-discrimination, accountability, participation, and the “AAAQ” framework (Figs. 4 and 5). Each of these seven principles is mentioned by more than 20 studies. The principle mentioned most often is accountability (30 studies). Transparency and redress, which are components of accountability, were mentioned in 15 and four studies respectively. In contrast, five studies do not mention any principle of the right to health. Eight studies mention the social determinants of health, usually an area of study in public health, as part of the right to health. Legal concepts guiding states’ obligations under the right to health were generally mentioned by fewer studies: core minimum of the right to health (10), international cooperation (7), and use of maximum available resources (6). The exception is the principle of progressive realisation which was mentioned by 19 studies. A glossary of the principles is provided in Additional file 2.

Public health scholars used outcome indicators, such as cancer incidence, cancer mortality, vaccination rates, infant and adult mortality rates and life expectancy. Two public health studies showed correlations between a health outcome indicator and one or several indicators intended to measure human rights outcomes, such as ratification of human rights treaties and the Human Development Index. One study used proxy indicators to measure human rights outcomes such as child labour, the Corruption Index or the Civil Liberties Index. Three public health studies used indicators aimed to understand health inequalities, such as the proportion of women who never received a cancer screening, the first course of treatment, waiting times and catastrophic health expenditures. They also proposed to disaggregate data by wealth quintiles, socio-economic status, urban/rural areas or sex to identify inequalities. Human rights scholars tended to use structural indicators such as constitutional provisions or domestic legislation recognising the right to essential medicines or the right to health, or the existence of a national medicines policy updated in the past 10 years. These scholars also used some outcome indicators, but these indicators were focused on the outcome of a right-to-health principle rather than a health outcome, e.g., vaccine quality, appropriateness of prescription of medicines, availability and pricing of essential medicines, availability of drug information leaflets in all common ethnic languages, and availability of medicines in prisons. These indicators measure the principles of quality and accessibility, including non-discriminatory access and the right to receive health information. Both human rights and public health scholars used macro-economic indicators, such as the proportion of Gross Domestic Product allocated to health, the proportion of health expenditure arising from out-of-pocket payments by individuals, and catastrophic health expenditures. All indicators are listed in Additional file 3.

A total of 39 international documents were mentioned. These include both human rights treaties and their General Comments - such as the Convention on the Rights of the Child or the Convention on the Elimination of All Forms of Discrimination against Women – and non-binding instruments such as the Universal Declaration of Human Rights, or global health agenda instruments such as the WHO Global Action Plan for the Prevention and Control of NCDs 2013-2020. The five instruments mentioned the most belong to both international human rights law and global health (Table 1). Three binding global health treaties were mentioned: the Framework Convention on Tobacco Control, the International Health Regulations, and the Agreement on Trade-Related Aspects of Intellectual Property Rights. Although these treaties do not strictly belong to the field of human rights, they have a considerable impact on the right to health, for example on the regulation of tobacco packaging or patent rights for essential medicines [21, 22].

**Table 1:** Five most mentioned international instruments by discipline

	Discipline			
	Human rights (8 studies)	Public health (37 studies)	Both disciplines (8 studies)	Total (53 studies)
International Covenant on Economic, Social and Cultural Rights	4 (50%)	19 (51%)	5 (63%)	28 (53%)
General Comment 14	4 (50%)	15 (41%)	5 (63%)	24 (45%)
Universal Declaration of Human Rights	5 (63%)	10 (27%)	3 (38%)	18 (34%)
WHO Constitution	5 (63%)	5 (14%)	3 (38%)	13 (25%)
Declaration of Alma-Ata	3 (38%)	5 (14%)	2 (25%)	10 (19%)
No document mentioned	2 (25%)	9 (24%)	1 (13%)	12 (23%)

Some studies referred to the UN Common Understanding of a human rights-based approach to development, the Sustainable Development Goals, national constitutions, and other national laws. In 12 studies, there was no reference to any instrument to underpin the assessment of the right to health.

Finally, we did not retrieve any studies from legal journals. We did find studies from the multidisciplinary Health and Human Rights Journal through reference searching. These papers helped to contrast how the right to health is discussed and assessed in the public health literature and the human rights literature.

## Discussion

The main aim of this research was to identify attempts to assess implementation of the right to health in the public health literature from 2000 to 2021. In the 53 studies included in our review, we observed five characteristics of such attempts: 1) studies on assessment of the right to health are recent and were first applied to the field of maternal health, HIV or TB; 2) principles of the right to health are not fully reflected in public health studies, which tend to focus on physical availability and accessibility of health medicines and services, as well as measures of health outcomes disaggregated by socio-economic groups; 3) Some public health studies present correlations between health outcomes and human rights, but the measures used for human rights are not targeted to the right to health; 4) the sources of the right to health are well known in public health; 5) our search did not retrieve legal studies, suggesting a lack of access to legal literature among public health scholars.

We found studies that were written by scholars from both human rights and public health disciplines. Whilst the public health community uses quantitative and qualitative methods to evaluate health programmes and policies, or to construct new indicators of the right to health, human rights studies largely focus on defining and discussing normative principles of the right to health, with the intent to construct indicators.

The fact that no relevant study was found before 2006 demonstrates a paucity of interest and information on the right to health within the public health literature until that time. The mandate of the first UN Special Rapporteur on the right to health (2002-08) and his numerous calls to develop indicators may be one reason for the sudden spark in research from 2006 to 2010 [23, 24]. He also called for the human rights and public health communities to work closely together to develop indicators of the implementation of the right to health, informed by international human rights principles and epidemiological methods [25, 26].

Studies published between 2006 and 2014 were mainly general, i.e., they discuss principles of the right to health, or the application of a human rights-based approach to health in practice. We make a parallel with the global agenda at that time and the calls of some scholars to include human rights dimensions in global targets, notably the Millennium Development Goals [26, 27].

The first studies on human rights-based approaches to health were focussed on maternal and child health [28–30]. From 2015, applications of human rights-based approaches to health became more varied, with studies on non-communicable diseases (chronic obstructive pulmonary disease in 2015, obesity in 2018, and cancer in 2019). This timeline corresponds to the primary focus of the global agenda on maternal health, HIV and TB during 2000-2010, and the shift to non-communicable diseases from 2013, with UN High-level Meetings on NCDs, and the WHO Global Action Plan for the Prevention and Control of NCDs 2013-2020 [31–34].

One study on universal health coverage was published in 2015, but this topic generated more interest from 2019, with four studies. This corresponds once again to the global agenda, with the focus on universal health coverage as target 3.8 of the Sustainable Development Goals for 2030 [35].

Principles of the right to health are often complex and difficult to translate in the public health field. The seven core principles that underpin the right to health were generally mentioned together, especially the AAAQ framework. However, eight public health studies did not mention any of these principles, which suggests that the right to health may have been applied as a label rather than used as the foundation for assessing health policies [18, 30, 36–41].

Some of these principles remain only partly understood. Some key legal components of the AAAQ framework are mentioned as principles of their own in the public health literature. Physical accessibility also tends to be mentioned as the only component of “accessibility”, whilst financial, informational, and non-discriminatory access are often mentioned as separate principles of the right to health.

The “acceptability” element of the AAAQ framework means that health services and drugs must respect cultural differences, and that health personnel must fulfil the obligation of patient confidentiality [2]. However, five studies referred to privacy and confidentiality as a right on its own, sometimes in addition to the AAAQ framework, showing once more the difference in the degree of understanding of the AAAQ framework between human rights and public health scholars.

Two concepts of the right to health appear less commonly in public health research: the concepts of the core minimum of the right to health, and international cooperation. States have core obligations that they must fulfil to realise the right to health. These obligations cannot legally be derogated from, even for economic reasons. They include the adoption of a national health strategy based on epidemiological evidence, or the prevention, treatment, and control of endemic and epidemic diseases [2]. Some of the core obligations are mentioned by public health scholars, such as the training of health personnel, access to information on health, the adoption of a national health strategy, and monitoring of health policies. However, these scholars do not refer to the legal value of these obligations, i.e., that they form part of the core minimum of the right to health, and therefore that when they are not fulfilled, this constitutes a direct violation of the right to health.

Further, states must also fulfil their obligation of international cooperation and assistance under the right to health, i.e., poorer states must seek aid if they do not have the resources to fulfil the right to health of their population, while richer states must provide financial and material resources to help those poorer states [2]. These core obligations and international assistance are almost exclusively discussed by human rights scholars, or by scholars attached to both fields. When assessing the right to health, public health scholars tend not to consider the obligations imposed upon states by international human rights law. Their primary focus is to measure some dimensions such as AAAQ, participation and non-discrimination, perhaps because those principles lend themselves better to quantitative and qualitative assessment.

Some public health scholars propose disaggregating data to understand health inequalities, while legal scholars and scholars from both disciplines tend to propose indicators to measure right-to-health principles that ensure inclusion of vulnerable groups. These studies proposed indicators to measure participation of affected populations and accessibility of health facilities, medicines and services on a non-discriminatory basis, for instance whether information is available in all common ethnic languages, or whether essential medicines are available to people in prisons. The AAAQ framework was more comprehensively assessed by scholars from both disciplines because public health scholars tend only to include measures of physical access. Some public health scholars selected indicators of the cause of poor health outcomes, such as the proportion of the population screened for a disease, first course of treatment and waiting times. Such indicators are not proposed by human rights scholars. Yet, if analysed in a disaggregated form, these indicators are critical to understanding which populations or sub-groups have access to prompt diagnosis and effective treatment. Some public health scholars correlate health outcomes with human rights outcomes, but the outcome indicators they use are not indicators of the right to health. These include measures of civil and political rights (as opposed to economic and social rights), such as political freedom, child labour and corruption. These indices have been criticised for their lack of transparency and their aggregation of several human rights into a single index [42]. A correlation between health outcomes and structural or process indicators of right-to-health principles would be more meaningful, because it would show whether laws and processes designed to implement the right to health do in fact result in better health outcomes and reduce health inequalities. Studies authored by experts in both human rights and public health included such structural and process indicators, as well as measures of participation, accountability and non-discrimination.

Regarding international instruments, there is a clear consensus among both the human rights and public health communities on the sources of the right to health. The most authoritative instruments that protect the right to health are the Covenant (Article 12), General Comment 14 which clarifies Article 12, the Universal Declaration of Human Rights (Article 25), and the WHO Constitution [1–4]. The Alma-Ata Declaration is also mentioned in most studies as a core instrument for a human rights-based approach to health [5]. However, nine public health studies do not mention any instrument in assessing the right to health, which reinforces the idea that the right to health may be used as the language of advocacy rather than as a formal basis of assessment.

Most of the studies we identified were published in public health journals. In legal journals, human rights indicators and the monitoring of economic and social rights, such as the right to health, have constituted a substantial body of research since the search for such indicators was initiated by the UN in 1993, then developed after 2000 [8]. Unless public health researchers also access legal journals, they may miss important articles that are relevant to the evaluation and monitoring of the right to health. For example, we found several key articles in legal databases that are influential in human rights research, such as research commissioned by the UN Development Programme in 2001, or the publication of the OPERA framework and the SERF index in 2012 [43–45]. We did not detect any article in the public health literature discussing the challenges in using indicators to monitor human rights, whereas this debate is significant in legal journals [46–49].

This observation suggests that there is a missing link between public health and legal scholars, apart from a few academics who publish in both fields. From a practical perspective, public health scholars may not have access to legal databases. They may thus be unaware of the comprehensive legal definition of the right to health, of the research conducted on human rights indicators over the past 30 years, and of the philosophical, moral, and ethical challenges that arise in developing such indicators. This point is echoed by experts who argue that human rights scholarship should be included in medical education and in public health institutions [22, 50].

## Conclusion

This scoping review shows that the right to health and human rights-based approaches to health are very recent topics in the public health literature, with the first studies published only in 2006. These approaches were first applied to maternal and child health, HIV or TB, but this diversified markedly from 2015, with new areas such as non-communicable diseases and universal health coverage, which corresponds to a shift in the global health agenda.

Some attributes of the right to health are clearly defined. These are non-discrimination, accountability, participation, and the AAAQ framework, even though the framework tends to be understood differently by the human rights and public health communities. These principles are generally translated into indicators more accurately by scholars who have expertise in both public health and human rights. Human rights scholars tend to use structural indicators to assess laws and policies, e.g., how compliant they are with the right to health and how well they are implemented in practice. International instruments that underpin the right to health are known by the public health community, even though some of the 53 studies we found did not refer to any such documents. Some studies seem to refer to the right to health for advocacy purposes rather than as a legal foundation of their method of assessment. Critically, our search did not retrieve any result from legal databases, which indicates that public health scholars have had very limited access to research conducted by legal scholars on human rights indicators and on monitoring of the right to health over the past 30 years.

Public health researchers who are interested in monitoring and evaluation of the right to health should expand their research to collaborate with legal scholars. The principles and legal foundations of the right to health seem fairly well known, but the challenges in monitoring the progressive realisation of this right, and the possibility of using these indicators to identify unjust laws and health policies, are less widely understood. Collaboration between the public health and human rights communities would allow an exchange of ideas, language and methods, and would significantly enrich research on the right to health.

## Supplementary Information

**Additional file 1.** Characteristics of studies. A table with all studies included in the scoping review as well as the data collected for each of them, i.e. year published; discipline (human rights, public health, or both); area of public health; type of research; international instruments referred to; principles of the right to health.

**Additional file 2.** Glossary. A glossary of the principles of the right to health used in this article.

**Additional file 3.** Studies with indicators used and analyses performed. A table with all studies that use indicators to assess implementation of the right to health, with description of the indicators used and analyses performed when relevant.

## Data Availability

The dataset supporting the conclusions of this article is included within Additional files 1 and 3.

## Abbreviations

AAAQ framework: Availability, Accessibility, Acceptability and Quality of health services and medicines
OHCHR: Office of the High Commissioner for Human Rights
The Covenant: International Covenant on Economic, Social and Cultural Rights
WHO: World Health Organisation
