# Supplementary material for: How should implementation of the human right to health be assessed? A scoping review of the public health literature from 2000 to 2021
Source: Int J Equity Health. 2022 Sep 22;21:139. doi: 10.1186/s12939-022-01742-0 (PMC9502920; doi:10.1186/s12939-022-01742-0)
Supplement: Supplementary file 1 — Additional file 1. Characteristics of studies. A table with all studies included in the scoping review as well as the data collected for each of them, i.e. year published; discipline (human rights, public health, or both); area of public health; type of research; international instruments referred to; principles of the right to health. [file 12939_2022_1742_MOESM1_ESM.pdf]

Additional file 1

Additional file 1. Characteristics of studies p.2

Full title of instruments in Additional file 1 p.21

Additional file 1. Characteristics of studies

Year published	Study	Journal	Area of public health	Type of research	Discipline public health, human rights, or both	International instruments used	Principles of the right to health
2006	Evans DP, Price ME, Gulrajani TL, Hinman AR. Making the grade: a first attempt at a health and human rights report card	Health and Human Rights Journal	General	Uses current public health indicators	Public health	UDHR<br>UN Charter<br>ICCPR<br>ICESCR	None
2006	Gruskin S. Rights-based approaches to health: something for everyone	Health and Human Rights Journal	General	Defines or discusses principles of the right to health	Human rights	None	Non exhaustive list:<br>● Participation<br>● Non-discrimination<br>● Legal and policy contexts<br>● Transparency<br>● Accountability
2006	Hogerzeil HV. Essential medicines and human rights: what can they learn from each other?	Bulletin of the WHO	Access to medicines	Defines or discusses principles of the right to health	Human rights	WHO Constitution<br>UDHR<br>Alma-Ata Declaration<br>ICCPR<br>ICESCR<br>General Comment 14	● Progressive realisation<br>● Immediate obligations<br>● Participation<br>● Accountability<br>● Transparency<br>● Equality and non-discrimination<br>● Redress
2007	Brennan F. Palliative care as an international human right	Journal of Pain Symptom Management	Palliative care	Defines or discusses principles of the right to health	Public health	ICESCR	● Access to health services and drugs on a non-discriminatory basis;<br>● Provision of essential drugs<br>● National public health strategy<br>● Progressive realisation<br>● Maximum available resources<br>● Duty-bearers (states)<br>● Enforceability

Year published	Study	Journal	Area of public health	Type of research	Discipline public health, human rights, or both	International instruments used	Principles of the right to health
2007	Gruskin S, Ferguson L, Bogecho DO. Beyond the numbers: using rights-based perspectives to enhance antiretroviral treatment scale-up	AIDS	HIV	Defines or discusses principles of the right to health	Public health	None	● Accountability<br>● Transparency of pricing of treatment<br>● Participation and advocacy<br>● Progressive realisation<br>● Monitoring with disaggregated data<br>● Non-discrimination<br>● AAAQ
2007	Hunt P. Right to the highest attainable standard of health	Lancet	General	Defines or discusses principles of the right to health	Both	WHO Constitution ICESCR General Comment 1	● Underlying determinants of health<br>● Non-discrimination<br>● Participation<br>● Accountability<br>● Progressive realisation

Year published	Study	Journal	Area of public health	Type of research	Discipline public health, human rights, or both	International instruments used	Principles of the right to health
2008	Backman G, Hunt P, Khosla R, et al. Health systems and the right to health: an assessment of 194 countries	Lancet	General	Constructs new indicators or a new framework	Both	ICESCR<br>CERD<br>WHO Constitution<br>Alma-Ata Declaration<br>Ottawa Charter<br>Bangkok Charter<br>General Comment 14<br>IHR<br>FCTC<br>General Comment 3<br>General Comment 4<br>CRC<br>General Recommendation 24<br>CEDAW<br>UDHR	● Legal recognition<br>● Participation<br>● Transparency<br>● Equity, equality, and non-discrimination<br>● Respect for cultural differences<br>● Planning<br>● Referral systems<br>● Coordination<br>● International cooperation<br>● Legal obligation<br>● Monitoring and accountability<br>● AAAQ

Year published	Study	Journal	Area of public health	Type of research	Discipline public health, human rights, or both	International instruments used	Principles of the right to health
2008	D'Ambruoso L, Byass P, Nurul Qomariyah S. Can the right to health inform public health planning in developing countries? A case study for maternal healthcare from Indonesia	Global Health Action	Maternal and child health	Reviews cases, programmes, policies, laws	Public health	UDHR<br>General Comment 14<br>Declaration on the right to development	● Availability: functioning facilities, goods, services, and programmes must be available in sufficient quantity<br>● Accessibility (non-discrimination): facilities, goods and services must be accessible to all, especially vulnerable and marginalised groups<br>● Accessibility (physical): facilities, goods and services must be within safe reach for all population (incl. marginalised groups)<br>● Accessibility (economic): facilities, goods and services must be affordable for all. Payment based on equity, whether public or private<br>● Accessibility (information): right to seek, receive and impart information on health issues<br>● Acceptability: facilities, goods and services must be respectful of medical ethics and culturally appropriate, sensitive to gender and respect confidentiality<br>● Quality: facilities, goods and services must be scientifically and medically appropriate

Year published	Study	Journal	Area of public health	Type of research	Discipline public health, human rights, or both	International instruments used	Principles of the right to health
2008	Hunt P, Backman G. Health systems and the right to the highest attainable standard of health	Health and Human Rights Journal	General	Defines or discusses principles of the right to health	Both	WHO Constitution<br>Alma-Ata Declaration<br>Ottawa Charter<br>General Comment 14	Principles must be applied to each of the 6 WHO building blocks of a health system.<br>● People-centred health systems<br>● Transparency, participation, equality and non-discrimination<br>● Respect for cultural differences<br>● Underlying determinants of health<br>● Progressive realisation<br>● Core obligations (such as planning)<br>● Quality<br>● Continuum of prevention and care with effective referrals<br>● Vertical or integrated approach<br>● Coordination<br>● International cooperation<br>● Striking balances<br>● Monitoring and accountability<br>● Legal obligations
2008	London L. What is a human-rights based approach to health and does it matter?	Health and Human Rights Journal	General	Defines or discusses principles of the right to health	Human rights	None	● Participation<br>● Information<br>● Redress<br>● Accountability: both states and private actors
2009	Gruskin S, Ferguson L. Using indicators to determine the contribution of human rights to public health efforts	Bulletin of the WHO	General	Defines or discusses principles of the right to health	Public health	None	● Participation<br>● Non-discrimination<br>● AAAQ<br>● Transparency<br>● Accountability
2009	Palmer A, Tomkinson J, Phung C, et al. Does ratification of human-rights treaties have effects on population health?	Lancet	General	Constructs new indicators or a new framework	Public health	UDHR<br>ICESCR<br>ICCPR<br>CRC<br>General Comment 14<br>Alma-Ata Declaration<br>CEDAW<br>CAT<br>CERD	● Progressive realisation<br>● Resources availability<br>● Core minimum obligations

Year published	Study	Journal	Area of public health	Type of research	Discipline public health, human rights, or both	International instruments used	Principles of the right to health
2010	Gruskin S, Bogecho D, Ferguson L. Rights-based approaches to health policies and programs: articulations, ambiguities, and assessment	Journal of Public Health Policy	General	Defines or discusses principles of the right to health	Both	CRC	● Duty-bearer: state's responsibility to respect, protect and fulfil<br>● Rights-holder<br>● Non-discrimination<br>● Participation<br>● Transparency<br>● Accountability: implies access to remedy<br>● Availability<br>● Accessibility (non-discrimination)<br>● Accessibility (physical)<br>● Accessibility (financial)<br>● Accessibility (information)<br>● Acceptability: incl. confidentiality<br>● Quality
2010	Rasanathan K, Norenhag J, Valentine N. Realizing human rights-based approaches for action on the social determinants of health	Health and Human Rights Journal	General	Defines or discusses principles of the right to health	Human rights	WHO Constitution<br>UDHR<br>ICESCR<br>Alma-Ata Declaration<br>General Comment 14	● Participation<br>● Equality<br>● Non-discrimination<br>● Accountability<br>● Rights holders and duty bearers<br>● Progressive realisation<br>● Social determinants of health
2010	Cottingham J, Kismodi E, Hilber AM, et al. Using human rights for sexual and reproductive health: improving legal and regulatory frameworks	Bulletin of the WHO	Sexual and reproductive health	Constructs new indicators or a new framework	Public health	None	None

Year published	Study	Journal	Area of public health	Type of research	Discipline public health, human rights, or both	International instruments used	Principles of the right to health
2010	Perehudoff SK, Laing RO, Hogerzeil HV. Access to essential medicines in national constitutions	Bulletin of the WHO	Access to medicines and constitutional provisions	Defines or discusses principles of the right to health	Public health	ICESCR General Comment 14	● Accessibility<br>●Availability<br>●Appropriateness<br>●Quality<br>of goods and services, incl. essential medicines as defined by the WHO Action Programme on Essential Drugs
2010	Chowdhury OH, Osmani SR. Towards achieving the right to health: the case of Bangladesh	The Bangladesh Development Studies	General	Reviews cases, programmes, policies, laws	Public health	Alma-Ata Declaration ICESCR	● Equity and non-discrimination<br>● Participation<br>● Accountability<br>● Progressive realisation<br>● AAAQ
2011	Mpinga EK, Chastonay P. Satisfaction of patients: a right to health indicator?	Health Policy	General	Defines or discusses principles of the right to health	Public health	General Comment 14 ICESCR	● Availability<br>● Accessibility (non-discrimination)<br>● Accessibility (physical)<br>● Accessibility (economic)<br>● Accessibility (information)<br>● Acceptability<br>● Quality
2011	Amin M, MacLachlan M, Mannan H, et al. EquiFrame: a framework for analysis of the inclusion of human rights and vulnerable groups in health policies	Health and Human Rights Journal	General	Constructs new indicators or a new framework	Public health	Alma-Ata Declaration	None

Year published	Study	Journal	Area of public health	Type of research	Discipline public health, human rights, or both	International instruments used	Principles of the right to health
2012	Williams C, Brian G. Using health rights to improve programme design: a Papua New Guinea case study	International Journal of Health Planning and Management	Eye health	Constructs new indicators or a new framework	Both	UDHR<br>ICESCR<br>General Comment 14	● AAAQ<br>● Progressive realisation<br>● Core obligations
2012	Gruskin S, Ahmed S, Bogecho D, et al. Human rights in health systems frameworks: what is there, what is missing and why does it matter?	Global Public Health	General	Defines or discusses principles of the right to health	Public health	ICESCR<br>General Comment 14	● AAAQ<br>● Equality and non-discrimination<br>● Participation<br>● Accountability<br>● Legal and policy contexts
2012	Maru D, Farmer P. Human rights and health systems development: confronting the politics of exclusion and the economics of inequality	Health and Human Rights Journal	General	Reviews cases, programmes, policies, laws	Public health	UDHR	● Participation, incl. marginalised groups<br>● Accountability of the state and its agents<br>● National public health strategy based on scientific evidence

Year published	Study	Journal	Area of public health	Type of research	Discipline public health, human rights, or both	International instruments used	Principles of the right to health
2013	Eide AH, Amin M, MacLachlan M, Mannan H, Schneider M. Addressing equitable health of vulnerable groups in international health documents	Alter	General	Constructs new indicators or a new framework	Public health	None	● Non-discrimination<br>● Individualised services<br>● Entitlement<br>● Capability-based services<br>● Participation<br>● Co-ordination of services<br>● Protection from harm<br>● Liberty<br>● Autonomy<br>● Privacy<br>● Integration<br>● Contribution<br>● Family resource<br>● Family support<br>● Cultural responsiveness<br>● Accountability<br>● Prevention<br>● Capacity building<br>● Access<br>● Quality<br>● Efficiency
2013	King EJ, Maman S, Wyckoff SC, Pierce MW, Groves AK. HIV testing for pregnant women: a rights-based analysis of national policies	Global Public Health	HIV	Reviews cases, programmes, policies, laws	Public health	None	● Consent: informed consent after receiving information on the risks of HIV testing<br>● Confidentiality<br>● Counselling<br>● Obligation to respect: evidence that governments are not violating the rights of pregnant women through national HIV testing policies (in particular with regards to consent and confidentiality)<br>● Obligation to protect: extent to which the policies address how to protect women from violence, stigma and other adverse consequences as a result of HIV testing and diagnosis<br>● Obligation to fulfil: provision of counselling services, access to treatment and plans for implementation

Year published	Study	Journal	Area of public health	Type of research	Discipline public health, human rights, or both	International instruments used	Principles of the right to health
2013	Heymann J, Cassola A, Raub A, Mishra L. Constitutional rights to health, public health and medical care: the status of health protections in 191 countries	Global Public Health	Constitutional provisions	Constructs new indicators or a new framework	Both	UDHR<br>CRC<br>CRPD<br>CERD<br>CEDAW<br>CRMW<br>ICESCR	● Interrelatedness of human rights<br>● Progressive realisation<br>● Transparency<br>● Accountability
2013	Uddin J, Momtaz S, Islam MS. State obligation towards the fulfillment of the right to health: a study in Bangladesh	Mediterranean Journal of Social Sciences	General	Reviews cases, programmes, policies, laws	Public health	National constitution<br>UDHR<br>ICESCR	● Underlying determinants of health, incl. access to information<br>● AAAQ, incl. access to information<br>● Monitoring and accountability<br>● Progressive realisation<br>● Non-discrimination<br>● Participation
2014	Hardee K, Kumar J, Newman K, Bakamjian L, Harris S, Rodriguez M, Brown W. Voluntary, human rights-based family planning: a conceptual framework	Studies in Family Planning	Maternal and child health	Constructs new indicators or a new framework	Public health	UDHR<br>ICESCR<br>General Comment 14	● Availability<br>● Accessibility<br>● Acceptability<br>● Quality<br>● Autonomy: principle of voluntarism, i.e. right to bodily integrity and security of person and right to decide freely and responsibly the number and spacing of children<br>● Information: right to sexual and reproductive health services, information, and education<br>● Equality and non-discrimination: right to make decisions concerning reproduction free of discrimination, coercion and violence<br>● Analysing health inequalities and laws and policies under which programmes operate<br>● Right holders and duty bearers<br>● Accountability<br>● Empowerment<br>● Non-discrimination<br>● Participation

Year published	Study	Journal	Area of public health	Type of research	Discipline public health, human rights, or both	International instruments used	Principles of the right to health
2015	Ranjbar V, Hjalmarsson A, Ascher H, Ekberg-jansson A. Chronic obstructive pulmonary disease mobile care: a participant-focussed and human rights-based evaluation	Health Services Management Research	Chronic obstructive pulmonary disease	Reviews cases, programmes, policies, laws	Public health	ICESCR	● AAAQ
2015	Sridhar D, McKee M, Ooms G, et al. Universal health coverage and the right to health: from legal principles to post-2015 indicators	International Journal of Health Services	UHC	Constructs new indicators or a new framework	Both	ICESCR General Comment 14	● Core minimum obligations as listed in General Comment 14, paragraph 43<br>● Progressive realisation, i.e. if a state does not provide healthcare or a service, it must demonstrate that it used all available resources<br>● Cost-effectiveness: states cannot prioritise expensive curative services which will be accessible only to a small portion of the population<br>● International assistance and cooperation: economic and technical<br>● Participatory decision-making<br>● Needs of vulnerable or marginalised groups
2015	Thomas R, Kuruvilla S, Hinton R, et al. Assessing the impact of a human rights-based approach across a spectrum of change for women's, children's, and adolescents' health	Health and Human Rights Journal	Maternal and child health	Defines or discusses principles of the right to health	Human rights	Common Understanding	● AAAQ<br>● Participation<br>● Equality and non-discrimination<br>● Accountability
2015	Hunt P, Yamin AE, Bustreo F. Making the case: what is the evidence of impact of applying human rights-based approaches to health?	Health and Human Rights Journal	General	Defines or discusses principles of the right to health	Both	None	None (editorial)

Year published	Study	Journal	Area of public health	Type of research	Discipline public health, human rights, or both	International instruments used	Principles of the right to health
2015	Yamin AE, Frisancho A. Human-rights-based approaches to health in Latin America	Lancet	General	Reviews cases, programmes, policies, laws	Public health	None	● Underlying determinants of health<br>● Participation<br>●Accountability (social and legal)<br>● Redress<br>● Transparency<br>● Equality and non-discrimination<br>● Duty-bearers and rights-holders
2016	Luh J, Cronk R, Bartram J. Assessing progress towards public health, human rights, and international development goals using frontier analysis	Plos ONE	General	Uses current public health indicators	Public health	None	● Progressive realisation
2016	Perehudoff K, Toebes B, Hogerzeil H. A human rights-based approach to the reimbursement of expensive medicines	Bulletin of the WHO	Access to medicines	Defines or discusses principles of the right to health	Public health	ICESCR<br>General Comment 3<br>Limburg Principles<br>TRIPS<br>Ruggie Principles	● Progressive realisation<br>● Maximum available resources<br>● Transparency<br>● Non-discrimination<br>● Accountability
2016	Perehudoff KS, Toebes B, Hogerzeil H. Essential medicines in national constitutions: progress since 2008	Health and Human Rights Journal	Access to medicines and constitutional provisions	Constructs new indicators or a new framework	Human rights	WHO Constitution<br>UDHR<br>ICESCR<br>General Comment 14	● Responsibility to respect, protect, and fulfil<br>● AAAQ of medicines-related rights<br>● Core obligation: immediate action on the provision of medicines

Year published	Study	Journal	Area of public health	Type of research	Discipline public health, human rights, or both	International instruments used	Principles of the right to health
2017	De Luca GB, Zopunyan V, Burke-Shyne N, Papikyan A, Amiryan D. Palliative care and human rights in patient care: An Armenia case study	Public Health Reviews	Palliative care	Reviews cases, programmes, policies, laws	Public health	General Comment 14	● Right to information regarding prognoses, treatment options and side effects in an easily understandable way<br>● Informed consent of patient before any intervention or treatment<br>● Right to privacy and confidentiality<br>● Training on palliative care for healthcare providers<br>● Removal of legal barriers to prescribe opioids to patients with life-long conditions
2017	Gruskin S, Ferguson L, Kumar S, Nicholson A, Ali M, Khosla R. A novel methodology for strengthening human rights based monitoring in public health: family planning indicators as an illustrative example	Plos ONE	Maternal and child health	Defines or discusses principles of the right to health	Public health	General Comment 20<br>General Comment 14<br>UN Recommendation 24<br>CEDAW<br>ICESCR<br>UNDP report 2005<br>General Comment 15<br>CRC<br>HRC report 2012	● Non-discrimination<br>● AAAQ<br>● Informed decision-making: full, prior, free, and informed consent<br>● Privacy and confidentiality<br>● Participation<br>● Accountability
2018	Jain AK, Hardee K. Revising the Family Planning Quality of Care Framework in the context of rights-based family planning	Studies in Family Planning	Maternal and child health	Defines or discusses principles of the right to health	Public health	General Comment 14<br>General Comment 22	● Availability<br>● Accessibility (physical, economic, information)<br>● Acceptability<br>● Quality: evidence-based practices that are scientifically and medically appropriate<br>● Information exchange: two-way communication between service providers and clients where clients have the right to seek information<br>● Right of the client to switch method of contraception or provider<br>● Dignity, respect to the person and confidentiality

Year published	Study	Journal	Area of public health	Type of research	Discipline public health, human rights, or both	International instruments used	Principles of the right to health
2018	Perehudoff SK, Alexandrov NV, Hogerzeil HV. Access to essential medicines in 195 countries: a human rights approach to sustainable development	Global Public Health	Access to medicines	Constructs new indicators or a new framework	Public health	Same as Backman et al (2008)	● Legal obligation to fulfil the right to health<br>● Use of maximum available resources<br>● Minimum core obligation to provide essential medicines<br>● Non-discrimination<br>● Progressive realisation: to expand access to health services, incl. essential medicines, using maximum available resources<br>● AAAQ<br>● Adoption of a national plan<br>● Transparency<br>● Participation<br>● Monitoring and accountability mechanisms
2018	Patterson D, Buse K, Magnusson R, Toebes B. Identifying a human rights-based approach to obesity for states and civil society	Obesity Reviews	Obesity	Defines or discusses principles of the right to health	Public health	2018 Political Declaration of the 3rd High Level Meeting on NCDs<br>WHO Constitution<br>ICESCR<br>UDHR<br>Global Action Plan for the Prevention and Control of NCDs 2013-2020<br>FCTC<br>CRC	● Accountability of duty-bearers<br>● Rights-holders<br>● Participation<br>● States obligations to respect, protect, and fulfil<br>● Use of UN accountability mechanisms

Year published	Study	Journal	Area of public health	Type of research	Discipline public health, human rights, or both	International instruments used	Principles of the right to health
2019	Bueno De Mesquita J. The universal periodic review: a valuable new procedure for the right to health?	Health and Human Rights Journal	General	Defines or discusses principles of the right to health	Human rights	WHO Constitution<br>UDHR<br>ICESCR<br>CRC<br>CEDAW<br>General Comments<br>Special Rapporteur's reports<br>Regional human rights treaties<br>National Constitutions<br>SDGs	● Social determinants of health
2019	Burkholder TW, Hill K, Hynes EJC. Developing emergency care systems: a human rights-based approach	Bulletin of the WHO	Emergency care	Defines or discusses principles of the right to health	Public health	WHO Constitution<br>UDHR<br>ICESCR<br>CRC<br>General Comment 14<br>National Constitutions<br>Alma-Ata Declaration<br>Ouagadougou Declaration<br>SDGs	● AAAQ applied to emergency care<br>● 6 core obligations integrated into the WHO building blocks: access, essential medicines, equitable distribution, national public health strategy.<br>● Respect, promote, and protect right to emergency care<br>● Progressive realisation

Year published	Study	Journal	Area of public health	Type of research	Discipline public health, human rights, or both	International instruments used	Principles of the right to health
2019	Da Mota Almeida Peroni F, Lindelow M, Oliveira De Souza D, Sjoblom M. Realizing the right to health in Brazil's Unified Health System through the lens of breast and cervical cancer	International Journal for Equity in Health	Cancer	Uses current public health indicators	Public health	WHO Constitution<br>OECD's framework for elements of good cancer care	● Quality of services
2019	Gianella C, Pesantes MA, Ugarte-Gil C, et al. Vulnerable populations and the right to health: lessons from the Peruvian Amazon around tuberculosis control	International Journal for Equity in Health	TB	Reviews cases, programmes, policies, laws	Public health	1993 Peruvian Constitution	● Acceptability<br>● Availability<br>● Affordability<br>● Adaptability<br>● Quality
2019	Nygren-Krug H. The right(s) road to universal health coverage	Health and Human Rights Journal	UHC	Defines or discusses principles of the right to health	Human rights	UDHR<br>WHO Constitution<br>Alma-Ata Declaration<br>Astana Declaration<br>General Comment 14<br>General Comment 24<br>Ruggie Principles	● International assistance<br>● Core minimum obligations, incl. non-retrogression<br>● Maximum available resources<br>● Equality and non-discrimination<br>● Transparency<br>● Participation<br>● Accountability<br>● AAAQ

Year published	Study	Journal	Area of public health	Type of research	Discipline public health, human rights, or both	International instruments used	Principles of the right to health
2019	Rehfuess EA, Stratil JM, Scheel IB, et al. The WHO-INTEGRATE evidence to decision framework version 1.0: integrating WHO norms and values and a complexity perspective	BMJ Global Health	General	Constructs new indicators or a new framework	Public health	WHO Constitution	● AAAQ<br>● Equality and non-discrimination<br>● Accountability<br>● Participation<br>● Transparency (mentioned under "equity" pillar and not human rights)<br>● Social determinants of health (mentioned under "societal implications" pillar and not human rights)
2019	Stangl AL, Singh D, Windle M, et al. A systematic review of selected human rights programs to improve HIV-related outcomes from 2003 to 2015: what do we know?	BMC Infectious Diseases	HIV	Reviews cases, programmes, policies, laws	Public health	None	● AAAQ<br>● Rights-holders and duty-bearers (although not understood as states)<br>● Non-discrimination<br>● Participation<br>● Empowerment<br>● Accountability<br>● Interrelatedness of human rights
2019	Tuomisto K, Tiittala P, Keskimaki I, Helve O. Refugee crisis in Finland: challenges to safeguarding the right to health for asylum seekers	Health Policy	UHC	Reviews cases, programmes, policies, laws	Public health	General Comment 14<br>UDHR<br>ICESCR<br>CRC<br>Finnish Constitution<br>Other national laws	None

Year published	Study	Journal	Area of public health	Type of research	Discipline public health, human rights, or both	International instruments used	Principles of the right to health
2019	Cros M, Cavagnero E, Alfred JP, et al. Equitable realization of the right to health in Haiti: how household data inform health seeking behavior and financial risk protection	International Journal for Equity in Health	UHC	Uses current public health indicators	Public health	SDGs	● Accessibility (financial)
2019	Perehudoff SK, Alexandrov NV, Hogerzeil HV. The right to health as the basis for universal health coverage: a cross-national analysis of national medicines policies of 71 countries	Plos ONE	Access to medicines	Reviews cases, programmes, policies, laws	Public health	ICESCR General Comment 14	● Core obligations<br>● Progressive realisation<br>● Maximum available resources<br>● Legal mandate<br>● Participation<br>● Transparency<br>● Monitoring<br>● Non-discrimination<br>● International assistance and cooperation<br>● Cost-effectiveness<br>● Duty-bearer<br>● Accountability<br>● AAAQ

Year published	Study	Journal	Area of public health	Type of research	Discipline public health, human rights, or both	International instruments used	Principles of the right to health
2019	Perehudoff SK, Alexandrov NV, Hogerzeil HV. Legislating for universal access to medicines: a rights-based cross-national comparison of UHC laws in 16 countries	Health Policy and Planning	Access to medicines	Reviews cases, programmes, policies, laws	Public health	ICESCR General Comment 14	● Core obligations<br>●Progressive realisation<br>● Maximum available resources<br>● Legal mandate<br>● Participation<br>● Transparency<br>● Monitoring<br>● Non-discrimination<br>● International assistance and cooperation<br>● Cost-effectiveness<br>● Duty bearer<br>● Accountability<br>● AAAQ
2020	Kantamaturapoj K, Kulthanmanusorn A, Witthayapipopsakul W, et al. Legislating for public accountability in universal health coverage	Bulletin of the WHO	UHC	Reviews cases, programmes, policies, laws	Public health	None	● Participation<br>● Accountability<br>● Legal mandate
2020	Perehudoff K. Universal access to essential medicines as part of the right to health: a cross-national comparison of national laws, medicines policies, and health system indicators	Global Health Action	Access to medicines	Constructs new indicators or a new framework	Public health	ICESCR General Comment 14	● Progressive realisation<br>● Cost-effectiveness<br>● Core obligations<br>● Transparency<br>● Participation<br>● Duty-bearer<br>● Legal mandate<br>● Accountability and redress<br>● Monitoring and evaluation<br>● International assistance and cooperation<br>● Non-discrimination

Full title of instruments

Short title	Full title
Alma-Ata Declaration	Declaration of Alma Ata on Primary Health Care (1978)
Astana Declaration	Astana Declaration on Primary Health Care (2018)
Bangkok Charter	The Bangkok Charter for Health Promotion in a Globalized World (2008)
CAT	Convention against Torture and Other Cruel, Inhuman or Degrading Treatment or Punishment (1984)
CEDAW	Convention on the Elimination of all forms of Discrimination Against Women (1979)
CERD	Convention on the Elimination of Racial Discrimination (1965)
Common Understanding	UN Common Understanding on a Human Rights-Based Approach to Development Cooperation (2003)
CRC	Convention on the Rights of the Child (1989)
CRMW	Convention on the Rights of Migrant Workers (1990)
CRPD	Convention on the Rights of Persons with Disabilities (2006)
FCTC	Framework Convention on Tobacco Control (2003)
General Comment 1	General Comment No. 1: Reporting by States Parties (1981)
General Comment 14	General Comment No. 14: The Right to the Highest Attainable Standard of Health (Art. 12 of the Covenant) (2000)
General Comment 15	General Comment No. 15: The Right to Water (Arts. 11 and 12 of the Covenant) (2003)
General Comment 20	General Comment No. 20: Non-discrimination in economic, social and cultural rights (art. 2, para. 2 of the International Covenant on Economic, Social and Cultural Rights) (2009)
General Comment 22	General Comment No. 22 on the Right to sexual and reproductive health (article 12 of the International Covenant on Economic, Social and Cultural Rights) (2016)
General Comment 24	General Comment No. 24 on State obligations under the International Covenant on Economic, Social and Cultural Rights in the context of business activities (2017)
General Comment 3	General Comment No. 3: The Nature of States Parties' Obligations (Art. 2, Para. 1 of the Covenant) (1990)
General Comment 4	General Comment No. 4: The Right to Adequate Housing (Art. 11 (1) of the Covenant) (1991)
HRC report 2012	Report of the Human Rights Council (2012)
ICCPR	International Covenant on Civil and Political Rights (1966)
ICESCR	International Covenant on Economic, Social and Cultural Rights (1966)
IHR	International Health Regulations (2005)
Limburg Principles	Note verbale dated 5 December 1986 from the Permanent Mission of the Netherlands to the United Nations Office at Geneva addressed to the Centre for Human Rights ("Limburg Principles") (1987)

Short title	Full title
Ottawa Charter	Ottawa Charter for Health Promotion (1987)
Ouagadougou Declaration	Ouagadougou Declaration on Primary Health Care and Health Systems in Africa (2008)
Ruggie Principles	United Nations Guiding Principles on Business and Human Rights (2011)
SDGs	Sustainable Development Goals: the 2030 Agenda for Sustainable Development (2015)
Special Rapporteur’s reports	Reports of the UN Special Rapporteur on the Highest Attainable Standard of Physical and Mental Health
TRIPS	Agreement on Trade-Related Aspects of Intellectual Property Rights (1994)
UDHR	Universal Declaration of Human Rights (1948)
UN Charter	Charter of the United Nations (1945)
UN Recommendation 24	CEDAW General Recommendation No. 24: Article 12 of the Convention (Women and Health) (1999)
UNDP report 2005	Human Development Report (2005)
WHO Constitution	Constitution of the World Health Organisation (1946)
