# Supplementary material for: How should implementation of the human right to health be assessed? A scoping review of the public health literature from 2000 to 2021
Source: Int J Equity Health. 2022 Sep 22;21:139. doi: 10.1186/s12939-022-01742-0 (PMC9502920; doi:10.1186/s12939-022-01742-0)
Supplement: Supplementary file 2 — Additional file 2. Glossary. A glossary of the principles of the right to health used in this article. [file 12939_2022_1742_MOESM2_ESM.pdf]

Additional file 2. Glossary of principles of the right to health

	**Definitions**
AAAQ	All health services, goods and facilities shall be available, accessible, acceptable and of good quality (AAAQ). The precise nature of these elements will depend on the conditions prevailing in a particular state.[1]
Available	Functioning public health and health-care facilities, goods and services, as well as programmes, have to be available in sufficient quantity within the state party. The precise nature will vary depending on numerous factors, including the state party's development level.[1]
Accessible	Health facilities, goods and services have to be accessible to everyone without discrimination, within the jurisdiction of the state party. It includes: non-discrimination; physical accessibility; affordability and information accessibility.[1] Other sources include accessibility on a non-discriminatory basis.[2]
Acceptable	All health facilities, goods and services must be respectful of medical ethics and culturally appropriate as well as being designed to respect confidentiality and improve the health status of those concerned.[1]
Good quality	Health facilities must be scientifically and medically appropriate and of good quality. This requires skilled medical personnel, scientifically approved and unexpired drugs and hospital equipment, safe and potable water, and adequate sanitation.[1]
Accountability	The right to health brings with it the crucial requirement of accessible, transparent, and effective mechanisms of monitoring and accountability. Those with right-to-health responsibilities must be held to account in relation to the discharge of their duties, with a view to identifying successes and difficulties; so far as necessary, policy and other adjustments can then be made. Examples of accountability mechanisms are:<br>1. Judicial, e.g., judicial review of executive acts and omissions<br>2. Quasi-judicial, e.g., national human rights institutions, human rights treaty-bodies<br>3. Administrative, e.g., human rights impact assessment<br>4. Political, e.g., parliamentary committees<br>5. Social, e.g., civil society movements<br><br>The accountability mechanism should exist at the national, regional (if available) and international levels. Rights-holders are also entitled to effective remedies when duty-bearers have failed to discharge their right-to-health obligations. These remedies may take the form of restitution, rehabilitation, compensation, satisfaction or guarantees of non-repetition.[1]
Duty-bearer and right-holder	States are the legal entities responsible for human rights (duty-bearers). They have obligations under international human rights law towards their citizens and sometimes towards citizens of other countries. Individuals have entitlements and freedoms and can claim them before a court (rights-holders). Business entities are neither duty-bearers nor rights-holders under international human rights law.
Economic, social, and cultural rights	Those human rights relating to the workplace, social security, family life, participation in cultural life, and access to housing, food, water, healthcare, and education. These rights contain dual freedoms: freedom from the state (for instance, freedom from eviction) and freedom through the state (e.g., receiving assistance from the state to access adequate housing). These rights are protected by the International Covenant on Economic, Social and Cultural Rights (1966).[3]
Equality and non-discrimination	An approach that aims to ensure that all persons are equal before the law and are entitled to the equal protection of the law without distinction based on race, colour, sex, language, religion, political or other opinion, national or social origin, property, birth, or other status. The term anti-discrimination implies a more proactive approach to tackling the causes and impacts of discrimination.[4]
General Comment No. 14 on the right to the highest attainable standard of health	Adopted by the Committee on Economic, Social and Cultural Rights (CESCR) in May 2000, provides an authoritative interpretation of contours and contents of the right to the highest attainable standard of health. CESCR is the body which monitors the International Covenant on Economic, Social and Cultural Rights and also publishes its interpretation of the provisions of the Covenant in the form of General Comments.[1]
Informed consent	The consent of a patient prior to an intervention or treatment after full disclosure of the risks and benefits.
Interdependence and interrelatedness	The realisation of one right depends on the realisation of other rights. For instance, the realisation of the right to health depends on the realisation of the right to water, housing, education, and work.[5]
International assistance and co-operation	Health is not just a matter of domestic laws, policies, and circumstances. The policies of other states, including in trade and development, as well as the policies of intergovernmental organisations such as the World Bank and the International Monetary Fund, also have a profound impact on health. The control of infectious diseases, the dissemination of health research, and so on, has an international dimension. In practice, the realisation of the right to the highest attainable standard of health is dependent upon international assistance and cooperation. The human rights responsibility of international assistance and cooperation is reflected in important international

	instruments, not least the Charter of the United Nations, the Universal Declaration of Human Rights, the International Covenant on Economic, Social and Cultural Rights, the Convention on the Rights of the Child and the Declaration on the Right to Development.[1]
Minimum core obligations	Under the International Covenant on Economic, Social and Cultural Rights, there are obligations considered to be of immediate effect to meet the minimum essential levels of each of the rights. They are called minimum core obligations. If a state fails to meet these because it does not have the resources, it must demonstrate that it has made every effort to use all available resources to satisfy, as a matter of priority, these core obligations. Even if a state has clearly inadequate resources at its disposal, the government must still introduce low-cost and targeted programmes to assist those most in need so that its limited resources are used efficiently and effectively.[3] For the right to health, minimum core obligations are listed in paragraph 43 of General Comment 14. They are supplemented by the "obligations of comparable priority" in paragraph 44.[5]
Participation	Active and informed participation of individuals and communities in decision-making that has a bearing on their health.[1] This principle goes hand in hand with transparency of laws, policies, and decision-making processes.
Progressive realisation	The concept of "progressive realisation" describes a central aspect of states' obligations in connection with economic, social, and cultural rights under international human rights treaties. At its core is the obligation to take appropriate measures towards the full realisation of economic, social, and cultural rights to the maximum of their available resources. The reference to "resource availability" reflects a recognition that the realisation of these rights can be hampered by a lack of resources and can be achieved only over a period of time. Equally, it means that a state's compliance with its obligation to take appropriate measures is assessed in the light of the resources—financial and others—available to it. Many national constitutions also allow for the progressive realisation of some economic, social, and cultural rights.[3]
"Right to health" and "right to the highest attainable standard of health" are used as convenient abbreviations for the more accurate formulations of the "right of everyone to the enjoyment of the highest attainable standard of physical and mental health"[1]	The right to health is a fundamental human right, including freedoms and entitlements. It does not mean "a right to be healthy"; the government cannot fully ensure good health as it is influenced by factors which are in whole or in part outside the state's control, such as individual susceptibility to ill-health and adoption of unhealthy lifestyles. However, the entitlements include the right to an effective and integrated health system, encompassing health care and the underlying determinants of health, e.g., access to education, safe water, sanitation, and food, which is responsive to national and local priorities, and accessible, available, acceptable and of good quality to all. Participation by the population in all health-related decision-making at the community, national and international levels is a component of the right to health. The right to the highest attainable standard of health is codified in numerous legally binding international and regional human rights treaties. The right is also enshrined in numerous national constitutions. The right to health, as with all human rights, is linked to other rights, such as right to life and freedom from discrimination.<br>States have duties to respect, protect, and fulfil the right to the highest attainable standard of health. These duties are equally applicable to medical care and the underlying determinants of health. The obligation to respect requires states to refrain from interfering directly or indirectly with the enjoyment of the right to health. The obligation to protect requires states to take measures that prevent third parties from interfering with the right to health. Finally, the obligation to fulfil requires states to adopt appropriate legislative, administrative, budgetary, judicial, promotional, and other measures towards the full realisation of the right to health.[1]
Transparency	Both the law-making process and legal, financial, and governmental information must be available for the public to consult freely. Transparency is needed to ensure participation.

1. Backman G, Hunt P, Khosla R, et al. Health systems and the right to health: an assessment of 194 countries. *Lancet* 2008; **372**(9655): 2047–85. Supplementary material.
2. World Health Organisation, Office of the High Commissioner for Human Rights. A human rights-based approach to health. Geneva, 2009.
3. Office of the High Commissioner for Human Rights. Factsheet No. 33: frequently asked questions on economic, social and cultural rights. 2008. https://www.ohchr.org/Documents/Issues/ESCR/FAQ%20on%20ESCR-en.pdf (Accessed on 27 August 2021).
4. UN High Commissioner for Refugees. Glossary. https://www.unhcr.org/47cfad9e2.pdf (Accessed on 27 August 2021).
5. UN Committee on Economic Social and Cultural Rights. General Comment No. 14: The Right to the Highest Attainable Standard of Health (Art. 12 of the Covenant). E/C12/2000/4; 11 August 2000.
