# Supplementary material for: How should implementation of the human right to health be assessed? A scoping review of the public health literature from 2000 to 2021
Source: Int J Equity Health. 2022 Sep 22;21:139. doi: 10.1186/s12939-022-01742-0 (PMC9502920; doi:10.1186/s12939-022-01742-0)
Supplement: Supplementary file 3 — Additional file 3. Studies with indicators used and analyses performed. A table with all studies that use indicators to assess implementation of the right to health, with description of the indicators used and analyses performed when relevant. [file 12939_2022_1742_MOESM3_ESM.pdf]

Additional file 3. Studies with indicators used and analyses performed

Year published	Study	Journal	Discipline public health, human rights, or both	Indicators and type of analyses performed
2006	Evans DP, Price ME, Gulrajani TL, Hinman AR. Making the grade: a first attempt at a health and human rights report card	Health and Human Rights Journal	Public health	Structural:<br>● Health expenditures as percent of Gross Domestic Product (GDP)<br>● Percent of births attended by trained staff<br>● Net primary school female enrolment ratio<br>Process:<br>● Access to prenatal care<br>● Percent of children immunized against Diphtheria, Tetanus and Polio (DTP)<br>Outcome:<br>● Adult (15-60 years) male mortality rate (per 1,000)<br>● Infant mortality rate<br>● Percent of population with access to potable water
2006	Hogerzeil HV. Essential medicines and human rights: what can they learn from each other?	Bulletin of the WHO	Human rights	● Does the national constitution, or any other national law, recognise the right to health? Are there laws which specify the government's responsibility in ensuring equitable access to essential medicines? Is there a national list of essential medicines updated in the last two years?<br>● Is there a national medicines policy updated in the last ten years? Were patients' organisations and rural communities consulted when the policy was developed?<br>● Does the national medicines policy describe the obligations of the various stakeholders? Are there baseline and target data on access to essential medicines against which progress can be measured?<br>● Are disaggregated access statistics available for boys, girls, women and men, and for urban and rural populations? Are essential medicines available in prisons? Are training materials and drug information leaflets available in all common ethnic languages?<br>● Are legal mechanisms available and have they been used to file complaints about lack of access to essential medicines?
2008	Backman G, Hunt P, Khosla R, et al. Health systems and the right to health: an assessment of 194 countries	Lancet	Both	List of 71 indicators of the right to health applied to health systems, organised under the following headings:<br>● Recognition of the right to the highest attainable standard of health<br>● Non-discrimination<br>● Health information<br>● National health plan<br>● Participation<br>● Underlying determinants of health<br>● Access to health services<br>● Medicines<br>● Health promotion<br>● Health workers<br>● National financing<br>● International assistance and cooperation

Year published	Study	Journal	Discipline public health, human rights, or both	Indicators and type of analyses performed
				● Additional safeguards<br>● Awareness raising about the right to the highest attainable standard of health<br>● Monitoring, assessment, accountability, and redress
2009	Palmer A, Tomkinson J, Phung C, et al. Does ratification of human-rights treaties have effects on population health?	Lancet	Public health	Association between ratification of human rights treaties and health outcomes and social indicators.<br>Health outcome indicators:<br>● HIV prevalence<br>● Infant mortality<br>● Child mortality<br>● Life expectancy<br>Social indicators:<br>● Child labour (from UNICEF)<br>● Human Development Index (HDI)<br>● Gender gap (from a World Economic Forum report)<br>● Corruption Index (from Transparency International)<br>● Civil liberties (from Freedom House)<br>● Political rights score (from Freedom House)
2010	Perehudoff SK, Laing RO, Hogerzeil HV. Access to essential medicines in national constitutions	Bulletin of the WHO	Public health	Recognition of access to medicines and health technology in national constitutions
2010	Chowdhury OH, Osmani SR. Towards achieving the right to health: the case of Bangladesh	The Bangladesh Development Studies	Public health	● Use of public health indicators disaggregated rural/urban, sex, wealth quintiles<br>● Maternal mortality is used as an indicator of discrimination against women in the health system
2011	Mpinga EK, Chastonay P. Satisfaction of patients: a right to health indicator?	Health Policy	Public health	Patient satisfaction surveys
2012	Williams C, Brian G. Using health rights to improve programme design: a Papua New Guinea case study	International Journal of Health Planning and Management	Both	36 indicators proposed to measure the AAAQ of the eye health programme in Papua New Guinea, progressive realisation and core obligations of the right to health in relation to this programme. Each indicator is a question with three possible answers: addressed fully, partially or not at all.

Year published	Study	Journal	Discipline public health, human rights, or both	Indicators and type of analyses performed
2013	Uddin J, Momtaz S, Islam MS. State obligation towards the fulfillment of the right to health: a study in Bangladesh	Mediterranean Journal of Social Sciences	Public health	● Number of hospital beds per person<br>● Proportion of the population with access to safe drinking water<br>● Proportion of the population with adequate sanitation (sealed latrines)<br>● Life expectancy<br>● Infant mortality/neonatal mortality<br>● Infant malnutrition / population malnutrition<br>● Infant stunting<br>● Proportion of underweight children aged 6-59 months<br>● Proportion of children immunized<br>● Number of HIV-related deaths<br>● Proportion of HIV-deaths among drug users<br>● Number of deaths or disabilities caused by tobacco<br>● Maternal mortality ratio<br>● Proportion of deliveries with skilled birth attendant<br>● Proportion of pregnant women with malnutrition and/or anaemia<br>● Nurse/population ratio<br>● Doctor/population ratio<br>● Density of medical workers rural/urban<br>● Absenteeism in health care<br>● Proportion of the population using publicly funded health system<br>● Total National Health Expenditure<br>● Expenditure for health per person per year<br>● Proportion of the population living below the poverty line<br>● Proportion of national income held by the poorest 20% population<br>● Proportion of national income held by the richest 20% population<br>● Proportion of for-profit health sector<br>● Proportion of informal health care providers<br>● National survey on corruption in hospitals (Household Corruption Survey)<br>● Share of foreign aid received<br>● Availability of health technologies<br>● Availability of training for health care workers<br>● Level of salaries for public health care workers<br>● Proportion of doctors remaining in position<br><br>Disaggregated as far as possible by rural/urban and per wealth quintiles

Year published	Study	Journal	Discipline public health, human rights, or both	Indicators and type of analyses performed
2015	Sridhar D, McKee M, Ooms G, et al. Universal health coverage and the right to health: from legal principles to post-2015 indicators	International Journal of Health Services	Both	Ten indicators for universal health coverage based on the right to health:<br>● The existence of a legal mandate for UHC in the country<br>● The extent of coverage in terms of depth (which services are covered)<br>● The extent of coverage in terms of breadth (who is insured) with attention to equity<br>● The extent of coverage in terms of height (what proportion of costs are covered) with focus on reduction in share of out-of-pocket payments for health care<br>● The commitment of adequate resources to deliver UHC with focus on percentage of gross national product for healthcare<br>● Cost-effectiveness with attention to equity<br>● International assistance as a percentage of GDP<br>● Existence of an international development policy explicitly including specific provisions to promote and protect the right to health<br>● SARA assessment on participatory decision making<br>● SARA assessment on prioritisation of marginalised groups
2016	Perehudoff KS, Toebes B, Hogerzeil H. Essential medicines in national constitutions: progress since 2008	Health and Human Rights Journal	Human rights	WHO's Mid-Term Strategic Plan 2008-2013 indicators:<br>● Constitutional provision and/or domestic legislation recognizing the right to essential medicines (structural indicator)<br>● Regulatory capacity (process)<br>● Vaccine quality, prescribing appropriateness, and essential medicines availability and price (outcome)<br>Backman et al (2008) indicators:<br>● National policy on medicines and essential medicines list (structural)<br>● Public per capita spending on pharmaceuticals (process)<br>● Rate of immunization coverage (outcome)
2018	Perehudoff SK, Alexandrov NV, Hogerzeil HV. Access to essential medicines in 195 countries: a human rights approach to sustainable development	Global Public Health	Public Health	● Constitutional commitment to medicines (structural)<br>● National medicines policy (structural)<br>● National essential medicines list (process)<br>NB: both indicators 2 and 3 can go beyond the simple yes/no answer and capture how rights-compliant these documents are.<br>● Government spending on pharmaceuticals (process)<br>NB: can be complemented by sub-indicator of equity that disaggregates spending by wealth quintile.<br>● Essential medicines availability in the public sector (outcome)<br>● Essential medicines availability in the private sector (outcome)<br>● National child immunisation rate for measles (outcome)<br>● National child immunisation rate for the third dose of DTP (outcome)
2019	Da Mota Almeida Peroni F, Lindelow M, Oliveira De Souza D, Sjoblom M. Realizing the right to health in Brazil's Unified Health System through the lens of breast and cervical cancer	International Journal for Equity in Health	Public health	Indicators for breast cancer and cervical cancer, disaggregated by state and socio-economic status when available:<br>● Cancer incidence<br>● Cancer mortality<br>● Human papillomavirus (HPV) vaccination rate<br>● Genetic testing for breast cancer<br>● Proportion of met screening needs

Year published	Study	Journal	Discipline public health, human rights, or both	Indicators and type of analyses performed
				● Number of pap smear collected compared to number of tests performed<br>● Proportion of women who never received a cancer screening<br>● Ecologic correlation between HDI and mammography coverage<br>● Cancer stage at diagnosis<br>● Waiting times (disaggregated by care in hospital and care in general practice pathways)<br>● First course of treatment<br>● Availability of oncologists, Computerised Tomography scans and Magnetic Resonance Imaging, by region
2019	Gianella C, Pesantes MA, Ugarte-Gil C, et al. Vulnerable populations and the right to health: lessons from the Peruvian Amazon around tuberculosis control	International Journal for Equity in Health	Public health	Comparison between urban and rural, or concentrated and dispersed populations for:<br>● Time for smear analysis and delivery of results to patient<br>● Time to treatment<br>● Structural indicators measuring factors putting people at risk of tuberculosis<br>● Structural indicators measuring factors that limit access to quality health services<br>● Adaptability of the national tuberculosis guidelines to rural settings<br>Disaggregated by ethnicity, place of residence, economic status
2019	Cros M, Cavagnero E, Alfred JP, et al. Equitable realization of the right to health in Haiti: how household data inform health seeking behavior and financial risk protection	International Journal for Equity in Health	Public health	● Out-of-pocket payments<br>● Catastrophic health expenditure<br>● Use of health care services in the past three months<br>Comparisons by wealth quintiles<br>● Determinants of health seeking behaviour: literacy, income, education, geographic location, number of household members, health insurance status, gender, children <4, older adult >65
2019	Perehudoff SK, Alexandrov NV, Hogerzeil HV. The right to health as the basis for universal health coverage: a cross-national analysis of national medicines policies of 71 countries	Plos ONE	Public health	Checklist of 12 criteria to assess the quality of national medicines policies, reflecting principles of the right to health: legal recognition, core obligation, transparency, participation, monitoring, accountability, AAAQ, use of maximum available resources, international assistance and cooperation, cost-effectiveness and non-discrimination.
2019	Perehudoff SK, Alexandrov NV, Hogerzeil HV. Legislating for universal access to medicines: a rights-based cross-national comparison of UHC laws in 16 countries	Health Policy and Planning	Public health	Checklist of 12 criteria to assess the quality of national UHC laws reflecting principles of the right to health: legal recognition, core obligation, transparency, participation, monitoring, accountability, AAAQ, use of maximum available resources, international assistance and cooperation, cost-effectiveness, non-discrimination.

Year published	Study	Journal	Discipline public health, human rights, or both	Indicators and type of analyses performed
2020	Perehudoff K. Universal access to essential medicines as part of the right to health: a cross-national comparison of national laws, medicines policies, and health system indicators	Global Health Action	Public health	● Constitutional commitment to medicines (structural)<br>● National medicines policy (structural)<br>● National essential medicines list (process)<br>NB: both indicators 2 and 3 can go beyond the simple yes/no answer and capture how rights-compliant these documents are.<br>● Government spending on pharmaceuticals (process)<br>NB: can be complemented by sub-indicator of equity that disaggregates spending by wealth quintile.<br>● Essential medicines availability in the public sector (outcome)<br>● Essential medicines availability in the private sector (outcome)<br>● National child immunisation rate for measles (outcome)<br>● National child immunisation rate for the third dose of DTP (outcome)
